# Supplementary figures and images for: Temporal novelty detection and multiple timescale integration drive Drosophila orientation dynamics in temporally diverse olfactory environments
Source: PLoS Comput Biol. 2023 May 11;19(5):e1010606. doi: 10.1371/journal.pcbi.1010606 (PMC10205008; doi:10.1371/journal.pcbi.1010606)

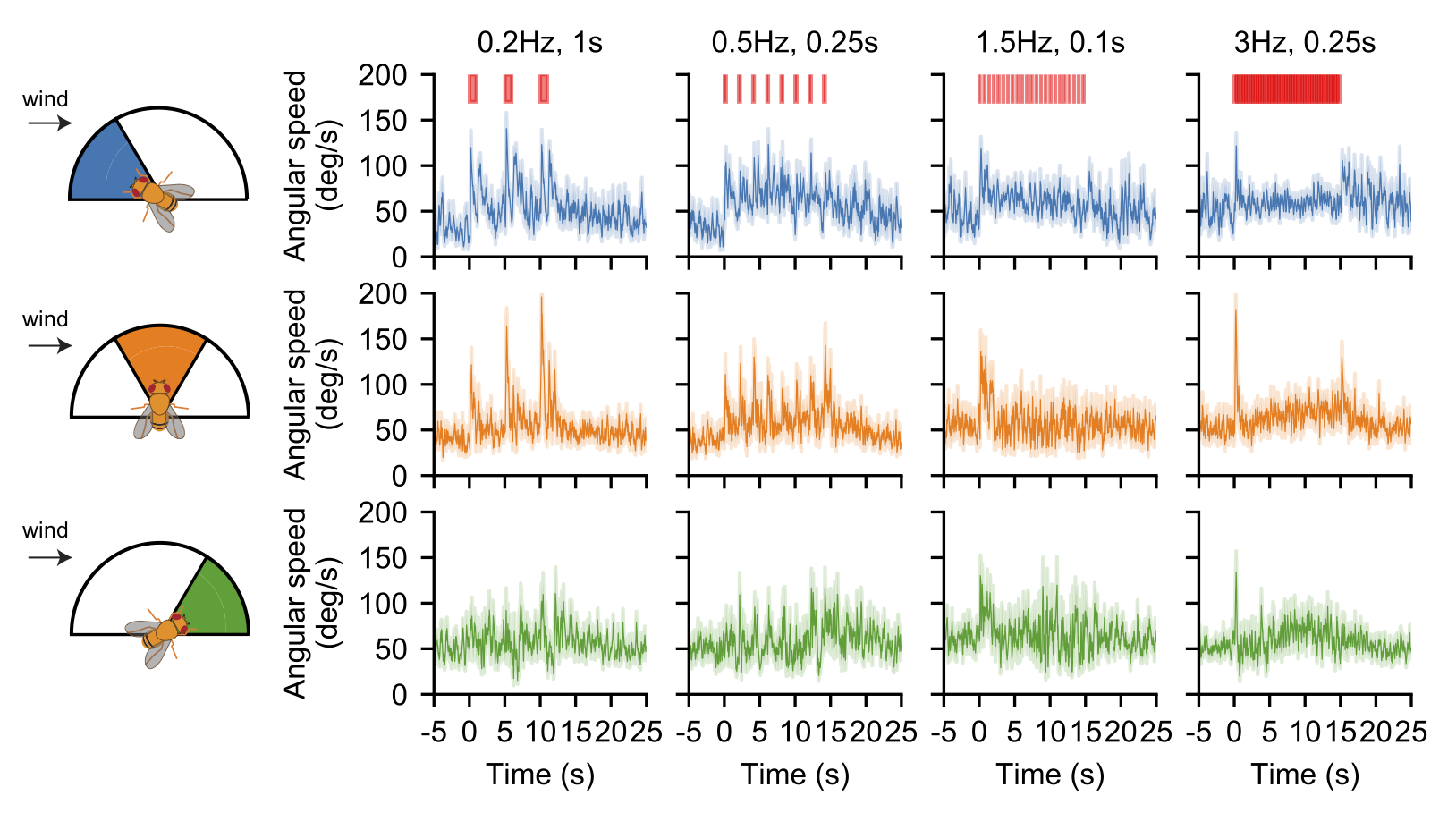

Supplement: S6 Fig — Heading was split into three bins of 60 degrees; upwind facing (120°-180°, blue), crosswind facing (60°-120°, orange) and downwind facing (0°-60°, green). Orientation was flipped over 180° as before. Solid line: population mean angular speed of flies oriented within the corresponding 60° bin at each time point. Lighter shading: mean ± SEM at each time point. Red bars: fictive odor pulses. Between 170 and 305 trajectories were recorded per environment for each 60° heading bin. Between 9 and 105 trajectories contribute to the data at each time point. (TIFF) [file pcbi.1010606.s006.tiff]
