# Supplementary material for: Temporal novelty detection and multiple timescale integration drive Drosophila orientation dynamics in temporally diverse olfactory environments
Source: PLoS Comput Biol. 2023 May 11;19(5):e1010606. doi: 10.1371/journal.pcbi.1010606 (PMC10205008; doi:10.1371/journal.pcbi.1010606)
Supplement: Attachment — Submitted filename: Reviewer_comments_responses_final.docx [file pcbi.1010606.s014.docx]

**Responses to the reviewer’s comments**

Reviewer #1: This manuscript by Sehdev…Emonet and colleagues builds on recent work from their group and others to further quantify the temporal cues in odor that drive olfactory navigation behaviors in Drosophila melanogaster. They use optogenetic activation of olfactory receptor neurons to examine behavioral responses to pulse odor stimuli of different frequencies and durations, and fit models to both the upwind turning component and a non-directed turn component evoked by onset and offset of the optogenetic stimulus. The main new findings are (1) a novelty non-directed turning response to a stimulus presented after a significant break, and (2) a refinement of a model from their previous paper (Jayaram et al) of the upwind turning response, arguing that a two-timescale integrator model fits the data somewhat better that the their previous model that linearly combines intermittency and frequency sensing. Overall, while I think the experiments and modeling are carefully done, I am concerned that the advance on their previous work is somewhat limited. In particular, the models presented in this paper do not incorporate the contribution of odor motion sensing that this group recently described, nor any possible contribution of spatial sensing to olfactory navigation (e.g. from Bhandawat lab work). Because they show model fits only to mean parameters of the behavioral data (e.g. turn rate or turn speed) and not simulations of full trajectories, it is not clear how significant the differences in the model from their previous models are for the ultimate navigation success of the animal, not whether the difference between the four models they propose for the temporal integration underlying upwind turning significantly exceeds the differences between individuals, which can be substantial. Although the scope of the paper is up to the authors, I feel that it would be more impactful for the field to put forward models that incorporate spatial and spatio-temporal contributions of odor sensing to navigation, in addition to temporal ones, and to make provision for understanding the role of individual differences in olfactory navigation behavior.

Major concerns:

1) The authors demonstrate the validity of their model with the ability recapitulate mean behavioral parameters (e.g. Figure 5D Fig. 6), but not he ability to capture individual variability or the trajectories of real flies. I think it would be useful to use their models to simulate real trajectories and evaluate whether the differences between this model and their/others previous models significantly impacts navigation success in different olfactory environments. In addition, it would be helpful to know which parameters of the model(s) account for differences across individuals. Several figures illustrating variation across individuals are unclear (see note on Fig. 1C below).

Following the reviewer’s suggestion we have now simulated agents executing our model while navigating simulated odor plumes. We have then examined how navigational performance varies depending on which of the models’ components are incorporated in the agent. Interestingly, we find that temporal novelty detection is most useful in conjunction with odor motion sensing. We have added a new paragraph and a new figure 7 to the main text describing these results. We thank the reviewer for this great suggestion. See also our response to point 2) below.

Regarding variation between individuals, due to the nature of our tracking we did not get sufficiently long trajectories to do a detailed analysis of individual behavior. We agree that investigating how the features and parameters of our model vary between individuals would be an interesting avenue for future study. This however would require much longer uninterrupted individual fly trajectories than we could obtain here. We have now amended our discussion to address (Lines 728-735).

2) The authors make several references to their exciting recent work showing the contribution of odor motion to navigation but odor motion effects on navigation do not form a part of the models presented here. How does odor motion sensing combine with the temporal sensing described here and what are the relative weights of these different factors? It seems like they should be able to address this using existing data from this and the previous paper.

Thank you for encouraging us to investigate how odor motion sensing interacts with the features in our model. While we wish to save a thorough experimental analysis for a future study, we have now investigated with simulation how motion sensing might be incorporated with the features of our model and what effect this has on navigation. The new results are summarized at the end of our results section and in a new Figure 7.

3) The use of optogenetic activation to produce navigation behaviors in flies is quite well established at this point (e.g. Bell…Wilson, Schulze..Louis, Tao…Bhandawat, Tumkaya…Clardig-Chang, Fox…Nagel, Matheson…Nagel), so I think the material in Figure 1 could be gone through more quickly.

We agree that optogenetic activation is well established and appreciate the value of brevity. However, we endeavoured to describe clearly our stimuli and to demonstrate that our results are due to optogenetic stimulation and not from any other confounding factors, and thus we would like to maintain the detail used in describing Figure 1.

4) The Discussion focusses almost exclusively on the adult antennal lobe, and does not mention significant experiments and models in the larval antennal lobe (e.g. Schulze…Louis, Gepner…Gershow) which likely performs similar temporal processing, nor work on central circuits for navigation in both larvae and adults (e.g. Tastekin…Louis, Matheson…Nagel) which could present additional loci for the temporal computations they identify here.

Thank you for your suggestion. We have now included them in the discussion.

additional:

Fig. 1C: I am confused by this plot which I think shows mean orientation. Why are the error bars not larger during the baseline (pre-odor) phase compared to odor? This figure seems to imply that all flies are fairly well oriented around 90° in the absence of stimulus, to the same degree that they are oriented at 135° during odor, which seems implausible to me. Are the flies shown here selected for a particular pre-odor orientation? Otherwise perhaps a histogram of orientations during the different phases would help to show variation in behavior across individuals.

Our results are similar to those from Demir et al (2020), which also found a similar variance between the pre and during odor blocks. Below are the histograms of the mean orientation of all trajectories during the ON and OFF blocks for the data in Figure 1C. There is a similar variance in the orientation for both the pre-odor and during-odor blocks (ON block variance: 1494.5, OFF block variance: 1245.4). Note that variable numbers of flies are recorded per time point, which will affect the SEM calculation. For example, at a given time point 5s into the ON block for the case shown in 1C, we have 113 trajectories contributing, with a mean heading of 129.7 degrees and standard deviation of 45.5 degrees. 5s into the OFF block we have 108 trajectories contributing with a mean heading of 87.0 degrees and standard deviation of 50.4 degrees (very similar to what we would expect from a uniform 0-180 degree distribution). Note how the similar standard deviations and trajectory numbers lead to similar SEMs in the ON and OFF block.

line 145: gmr-hid. Can you please spell out what this is and what it does?

Gmr-hid drives apoptosis (Grether et al 1995 Genes & Development) in the developing eye in *Drosophila* (Ellis et al 1993 Development). Thus *Drosophila* mutants with a copy of this gene are blind/have severely reduced eye function. We endeavoured to explain this in the Materials and Methods section, and have now updated this section with a more detailed description of gmr-hid.

line 159-165. This paragraph should note the many other studies that have used opto-genetic stimulation to evoke navigation behaviors (Tao/Bhandawat, Tumkaya/Gepner, Matheson/Nagel).

Thank you for highlighting several other important studies that use optogenetic stimulation when studying navigation. We have updated our references accordingly.

line 266: angular speed dynamics depended more on changes in turn rate and turn speed…”. This would seem to conflict with the statement in Demir et al that navigation does not depend on changes in turn rate. please comment.

Our original text was unclear. This apparent discrepancy is clarified in Figure 4A where we plot turn rate and turn speed as a function of time for environments with different odor pulse frequency. To make this clearer to the reader we added the following paragraph in the main text were we discuss Figure 4A (a few sentences below the one mentioned by the reviewer): “In low frequency signals, turn rate and turn speed appeared strongly modulated by each odor encounter (Figure 4A left four panels**)**, as previously seen in experiments that used real odors pulses of low frequency (Alvarez-Salvado et al., 2018). In contrast, in high frequency signals, turn rate and turn speed responded much more strongly to the first and last pulse of each block, while being only weakly modulated by individual odor pulses within the blocks (Figure 4A right four panels**)**, as previously observed in flies navigated complex plumes where odor encounters continuously occurred at high frequency (Demir et al., 2020).“

upwind turning model in Fig. 5B/C. For similarity with others literature, it might be helpful to plot this with the upwind point (0/180°) at the middle of the plot, illustrating that it forms a stable fixed point of the system. This would connect with literature on fixation in the visual system (e.g. Reichardt and Poggio, 1976) and recent studies in the central complex (e.g. Hulse et al. 2020, Goulard and Webb, 2021, Matheson/Nagel 2022).

In all of our figures angles are plotted between 0°-180° with 0° corresponding to downwind and 180° to upwind. In the paper mentioned by the reviewer angles are plotted over the full 0-360° range, and thus are symmetric across the downwind-upwind axis. Since we remove this symmetry in our plots, we believe that our current arrangement with crosswind in the middle makes the trend in upwind bias clearer and more interpretable for orientations between 0°-180°.

line 585: “for a simple integrating response to increase independently with frequency and intermittency, it is necessary for the rise timescale to be faster than the decay timescale” — doesn’t this have to be true?

Thank you for highlighting this potentially misleading phrase. We were attempting to say that for a two-timescale integrating response, the asymmetry in timescales enables independent response to both frequency and intermittency. Furthermore, a rapid rise and slower decay enables the response output to increase independently with both intermittency and frequency, as we show in the Materials and Methods section of this paper. We have amended the text to clarify this.

line 604: “we did not factor changes in ground speed or transitions between stops and walk bouts”. These have both been modeled before by this group and others—-why not incorporate these into the model?

In this paper we wanted to focus solely on orientation dynamics. In response to the reviewer’s comment we plotted the average walking speed and walking and stopping transition rates in a new supplemental Figure S1 and discuss these results in lines 741-752.

Reviewer #2: This is an elegant study, and I am very enthusiastic about the manuscript and its results. I only have one minor comment/question for the authors. In many moth species, the flux of the odor molecules has been shown to be critical for odor-mediated navigation. The optogenetic activation of the orco pathway in Drosophila allows precise control of the temporal dynamics of odor "encounter", but I'm wondering in this study if the authors have examined the interplay between concentration~encounter. The authors' eLife article touches on this aspect, but I'm wondering if it's possible to also examine this by scaling the temporal dynamics of the photostimulation with the intensity of the light stimulation as a proxy for odor concentration.

Thank you for this suggestion. It is an interesting idea worth examining. We are building a new setup with a projector that has higher spatial resolution and refresh rate. Once this system is ready, we should be able to study this.

**Have the authors made all data and (if applicable) computational code underlying the findings in their manuscript fully available?**
The [PLOS Data policy](https://nam12.safelinks.protection.outlook.com/?url=https%3A%2F%2Fjournals.plos.org%2Fploscompbiol%2Fs%2Fmaterials-and-software-sharing&data=05%7C01%7Caarti.sehdev%40yale.edu%7C7c626495b5bd4e60d87b08dad6d7ac21%7Cdd8cbebb21394df8b4114e3e87abeb5c%7C0%7C0%7C638058519058784103%7CUnknown%7CTWFpbGZsb3d8eyJWIjoiMC4wLjAwMDAiLCJQIjoiV2luMzIiLCJBTiI6Ik1haWwiLCJXVCI6Mn0%3D%7C3000%7C%7C%7C&sdata=FcYfIFRdkPwrl%2F0yWjuQBnPa269wt8TgeiLOus%2BaPoA%3D&reserved=0) requires authors to make all data and code underlying the findings described in their manuscript fully available without restriction, with rare exception (please refer to the Data Availability Statement in the manuscript PDF file). The data and code should be provided as part of the manuscript or its supporting information, or deposited to a public repository. For example, in addition to summary statistics, the data points behind means, medians and variance measures should be available. If there are restrictions on publicly sharing data or code —e.g. participant privacy or use of data from a third party—those must be specified.

Reviewer #1: **No:** manuscript says they are "available upon request"

Reviewer #2: **No:** The authors should place their code and data on github (immediately after the manuscript is accepted)
