# Supplementary material for: Temporal novelty detection and multiple timescale integration drive Drosophila orientation dynamics in temporally diverse olfactory environments
Source: PLoS Comput Biol. 2023 May 11;19(5):e1010606. doi: 10.1371/journal.pcbi.1010606 (PMC10205008; doi:10.1371/journal.pcbi.1010606)
Supplement: Attachment — Submitted filename: Reviewer_Response.docx [file pcbi.1010606.s015.docx]

We thank the reviewers for reading our revised manuscript in details and for providing feedback.

A few minor points to add to the Discussion:

— Although the authors use optogenetic activation of orco to dissect the temporal dynamics of “odor” that drive components of behavior, I think there is strong evidence that different odors are processed differently in time to drive behavior. This was recently demonstrated elegantly by Zocchi…Hong (Current Biology, 2022) who showed that flies will walk upwind in a continuous stream of apple cider vinegar, but require pulse CO2 to show an upwind behavioral response. This is consistent with earlier findings in mosquitoes that different dynamics of human scent and CO2 drive approach behavior. I think this point should be included in the Discussion or Intro.

Thank you for the suggestion. We added a few sentences discussing this starting at line 762.

— another recent preprint (Aso…Hige, bioRxiv, 2022) identifies additional central circuitry that plays a part in upwind orientation behavior.

We also mentioned in the same location the role of memories and cited Aso et al.

minor things:

line 29: maybe “the fraction”?

Updated as suggested.

line 59: “incredibly challenging task” I dunno. Ermentrout has done some work showing that a variety of relatively simple algorithms can be successful in certain plume structures. Maybe just “challenging”?

Updated as suggested.

line 84: “straight odor ribbons” not all of the cited papers used ribbons of odor.

We have removed the mention of straight odor ribbons.

line 93: frequency of odor encounters. see my point above about the Zocchi paper. I think it is unlikely that there is a single set of dynamics that all insects use to process odor, rather the dynamics of odor that evoke behavior is likely odor and species specific.

Rather than modifying this phrase we added a mention about the role of odor identity in the previous sentence (Line 91): “[…] wind, along with odor gradients and the recently-discovered odor motion, indicate directions of interest, while the timing of odor encounters and the odor identity indicate when to turn and how much to bias the new orientation in the directions of interest.”

line 200: “Combination that produced indeterminable encounters were excluded.” I am not sure what you mean by this.

We meant that since we use a fixed sets of duration and frequencies, some intermittencies are not explored in this set of experiments. This sentence was just confusing. We removed it.

line 487: “For the intermittency sensing model (…) predicted well the response. This sentence is not grammatical.

Fixed.

Reviewer #2: The authors have performed an excellent study, and I am excited to see it published.

Thank you for the supporting feedback.
